# Supplementary material for: Development of a computer-aided design software for the quantitative evaluation of aesthetic damage
Source: PLoS One. 2019 Dec 18;14(12):e0226322. doi: 10.1371/journal.pone.0226322 (PMC6919621; doi:10.1371/journal.pone.0226322)
Supplement: S1 Code site — 1. (ZIP) [file pone.0226322.s001.zip › [EESC_jr][Projeto]Relatorio_07_21/vendors/select2/docs/_includes/options/selections/templating.html]

## How can I customize the way selections are displayed?

When a selection is made by the user Select2 will display the text of the option by default, just like how it is displayed in a standard select box. You can override the display of the selection by setting the `templateSelection` option to a JavaScript function.

{% highlight js linenos %}
function template(data, container) {
return data.text;
}
$('select').select2({
templateSelection: template
});
{% endhighlight %}

### Nothing is being displayed when I select an option

{% include options/not-written.html %}

### I am using HTML in my selection template but it isn't displaying it

{% include options/not-written.html %}

### How can I access the container where the selection is displayed?

{% include options/not-written.html %}
